# Genome-wide identification of lineage and locus specific variation associated with pneumococcal carriage duration

**DOI:** 10.7554/eLife.26255

**Published:** 2017-07-25

**Authors:** John A Lees, Nicholas J Croucher, David Goldblatt, François Nosten, Julian Parkhill, Claudia Turner, Paul Turner, Stephen D Bentley

**Affiliations:** 1 Infection GenomicsWellcome Trust Sanger InstituteHinxtonUnited Kingdom; 2 Department of Infectious Disease EpidemiologySt. Mary’s Campus, Imperial College LondonLondonUnited Kingdom; 3 Institute of Child HealthUniversity College LondonLondonUnited Kingdom; 4 Shoklo Malaria Research Unit, Mahidol-Oxford Tropical Medicine Research Unit, Faculty of Tropical MedicineMahidol UniversityMae SotThailand; 5 Centre for Tropical Medicine and Global Health, Nuffield Department of MedicineUniversity of OxfordOxfordUnited Kingdom; University of ChicagoUnited States

**Keywords:** *S. pneumoniae*, GWAS, carriage duration, epidemiology, heritability, Other

## Abstract

*Streptococcus pneumoniae* is a leading cause of invasive disease in infants, especially in low-income settings. Asymptomatic carriage in the nasopharynx is a prerequisite for disease, but variability in its duration is currently only understood at the serotype level. Here we developed a model to calculate the duration of carriage episodes from longitudinal swab data, and combined these results with whole genome sequence data. We estimated that pneumococcal genomic variation accounted for 63% of the phenotype variation, whereas the host traits considered here (age and previous carriage) accounted for less than 5%. We further partitioned this heritability into both lineage and locus effects, and quantified the amount attributable to the largest sources of variation in carriage duration: serotype (17%), drug-resistance (9%) and other significant locus effects (7%). A pan-genome-wide association study identified prophage sequences as being associated with decreased carriage duration independent of serotype, potentially by disruption of the competence mechanism. These findings support theoretical models of pneumococcal competition and antibiotic resistance.

## eLife digest

Microorganisms live in most parts of our body, including the inside of our nose. Most of the microbes are harmless and can even be beneficial to our health. However, some microbes can cause diseases – although they often go unnoticed, as our immune system can remove them before we show any symptoms. For example, the bacterium *Streptococcus pneumoniae* can cause diseases such as pneumonia and meningitis, but generally, it lives harmlessly in the nose, and is particularly common in children and the elderly.

The longer the bacteria live in the nose before being killed by the immune system, the more likely they are to be transmitted to another person. The amount of time it takes for the immune system to clear the bacteria depends on various factors, such as the age of the person or the bacterium’s defense mechanism and its genetic material. A particularly important aspect is to what subtype, also known as serotype, a bacterium belongs to, which is characterized by differences in the structure of the sugar coating that surrounds the microbe. However, until now, it was not known how much each of these factors contributes.

Now, Lees et al. have developed a mathematical model to calculate how long the bacteria are carried in the nose before they are cleared away, and compared it with the genomic data of the bacteria. For this, over 14,000 nose swabs from almost 600 children were collected over a two-year period. In their model, Lees et al. calculated that the bacteria’s genetics explained over 60% of the variability in survival time. They also found that the serotype was the most important individual factor that influenced how long a bacterium could survive. The age of the child was less important and only accounted for 5%. In addition, Lees et al. also found that when viruses infected some *S. pneumoniae*, the bacteria died sooner.

A next step will be to confirm the effect of a viral infection on the bacteria’s survival time in a controlled model system, and also replicate the findings in separate population study.Understanding how long people can carry bacteria and transmit them to others may help to develop new vaccination or treatment strategies to control infections. Moreover, the discovery that viruses can negatively affect how long a bacterium lives, could motivate studies to investigate these findings further.

## Introduction

*Streptococcus pneumoniae* is a human pathogen that can cause diseases such as pneumonia, otitis media and meningitis. Pneumococcal disease burden is highest in children (O'Brien et al., 2009). For disease to be caused pneumococci must first transmit to the host, colonise the nasopharynx and finally cross into a normally sterile site. The pneumococcus spends most of the transmission cycle in the nasopharynx, and so understanding and predicting the amount of time spent in this niche is critical for understanding this bacterium's epidemiology, and therefore controlling transmission (Abdullahi et al., 2012a; Melegaro et al., 2007).

The nasopharynx is a complex niche in which each pneumococcal genotype must tackle a wide range of factors including host immune defence (McCool et al., 2002), other bacterial species (Pericone et al., 2000), and other pneumococcal lineages (Auranen et al., 2010; Cobey and Lipsitch, 2012) in order to maintain the genotype's population. The average nasopharyngeal duration period is therefore affected by a large number of factors, which may, themselves, interact.

One factor that is known to strongly associate with carriage duration is serotype: as capsular polysaccharides are important in bacterial physiology and determining host immune response, different serotypes have different clearance and acquisition rates (Abdullahi et al., 2012a; Hill et al., 2010; Högberg et al., 2007; Melegaro et al., 2004; Turner et al., 2012). Additionally, a range of other proteins have been identified as critical to the colonisation process (Kadioglu et al., 2008), some of which exhibit similar levels of diversity to the capsule polysaccharide synthesis locus (Iannelli et al., 2002; Jedrzejas et al., 2001). However, the overall and relative contributions of these sequence variations to carriage rate have not yet been characterised. In addition variation of pathogen protein sequence, accessory genes and interaction effects between genetic elements may also have as yet unknown effects on carriage duration.

Changes in average carriage duration have been shown to be linked with recombination rate (Chaguza et al., 2016), which has been found to correlate with antibiotic resistance (Hanage et al., 2009) and invasive potential (Chaguza et al., 2016). The carriage duration by different serotypes is widely used in models of pneumococcal epidemiology, and consequently is important in evaluating the efficacy of the pneumococcal conjugate vaccine (PCV) (Melegaro et al., 2007; Weinberger et al., 2011). Additionally, modelling work has proposed that if alleles exist which alter carriage duration, these explain the long standing puzzle of how antibiotic-resistant and sensitive strains stably coexist in the population (Lehtinen et al., 2017). Measurement of carriage duration and the analysis of its variance beyond the resolution of serotype will have important consequences for these models.

**Figure 1.**
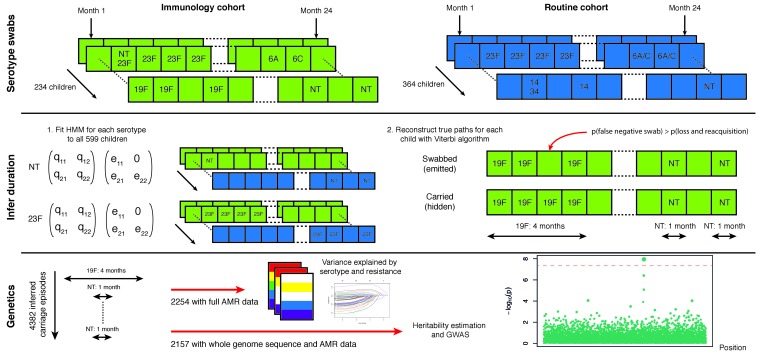
Swabbing and sequencing study design. We start with serotype swab data on 598 children from two cohorts, taken every month after birth for two years. For all samples we fitted the transition and emission probabilities of a continuous time hidden Markov model for each serotype. Then, for each child, we used these parameters were then used to infer the most likely carriage durations. We matched carriage episodes with resistance and genomic data for 2157 episodes to draw conclusions on the basis of variation in this epidemiological parameter.

We sought to determine the overall importance of the pathogen genotype in carriage duration in a human population, and to identify and quantify the elements of the genome responsible for the variation in carriage duration. By combining epidemiological modelling of longitudinal swab data with and genome wide association study methods on the connected sequences (Figure 1), we made heritability estimates for carriage duration. We further partitioned the heritability into contributions from lineage and locus effects (Earle et al., 2016) to quantify the variation caused by each individual factor.

## Results

### Ascertainment of carriage episode duration using epidemiological modelling

We first estimated carriage duration from longitudinal swab data available for the study population. For 598 unvaccinated children up to 24 swabs taken over a two year period were available, an extension on the previous study (Turner et al., 2012, 2013a). We only considered swabs from infants in the study, as mothers did not have sufficient sampling resolution relative to their average length of carriage to determine carriage duration. Furthermore, the immune response of mothers to bacterial pathogens is different to children (Maródi, 2006), leading to shorter carriage durations (Gritzfeld et al., 2014).

To estimate carriage duration from the longitudinal swab data we constructed a set of hidden Markov models (HMMs) with hidden states corresponding to whether a child was carrying a serotype at a given time point, and observed states corresponding to whether a positive swab was observed for this serotype at this time point.

The most general model for the swab data would be a vector with an entry of 0 or 1 for every possible serotype (of 56 observed in the population), corresponding to whether each serotype was observed in the swab at each time point. However, the number of parameters to estimate in this model (with over 6 million states) is much larger than the number of data points (around 14000), and in particular some serotypes have very few positive observations. Instead, we modelled each serotype separately.

**Figure 2.**
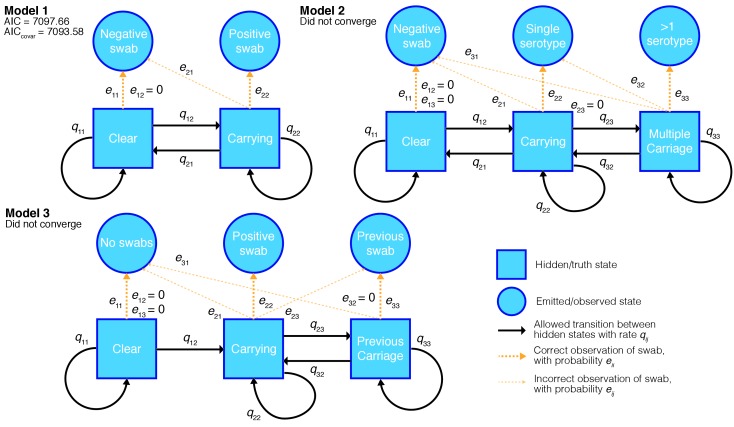
Hidden Markov models of swab time series, and their goodness-of-fit. We fitted three different models to the processed time-series data with states, allowed transitions and emissions as shown. We refitted each model allowing the transitions probabilities to covary with the age of the child and whether the child had carried pneumococcus previously. For the converged model the Akaike information criterion (AIC) is shown for the original fit, and when including these covariates (AICcovar).

The models fitted, and their permitted transitions and emissions are shown in Figure 2. In model one, observation i emits state 2 if positively swabbed for the serotype, and state 1 otherwise. The unobserved states correspond to the child ‘carrying’ and being ‘clear’ of the serotype respectively. We assume swabs have a specificity of one, so do not show positive culture when the child is clear of the carried serotype; we therefore set the coefficient for the chance of observing positive culture when no bacteria are present to zero ($e_{21}=0$ in the emission matrix). Model two adds a third state of ‘multiple carriage’ which is occupied when the serotype and at least one other are being carried. Both models were compared with a version which allows the parameters to covary with whether the child has carried pneumococcus previously. Model three accounts for this explicitly by having separate states and emissions based on whether carriage has previously been observed.

We applied all the models to 19F carriage episodes, as these had the most data available, and calculated the Akaike information criterion (Akaike, 1974) for each model that converged. Only the simplest model (model one) converged, as judged by having a positive-definite Hessian and a converged BOBYQA run. The more complex models had lower log-likelihoods: as extensions of the simpler model they should have higher log-likelihoods, so this results was not consistent with model convergence. We tried fitting models two and three using a fixed false positive values slightly greater than zero, this lead to better log-likelihoods, but the models still didn’t converge. This failure of the more complex models is probably because most children in the study immediately enter the carrying state, and episodes of dual carriage (when split up by serotype) are rare. Therefore there were not enough events between these carriage states to estimate to the transition and emission intensities, without sensitivity to initial conditions during the fitting.

We then fitted the best performing model in this test for all serotypes separately. 6A and 6C were treated as a single serotype, as they were not always distinguished in the course of the study. The models for 19F, 23F, 6A/C, 6B, 14 and non-typable (NT) converged, but other serotypes did not have enough observations to successfully fit the parameters of the model. For these less prevalent serotypes we used the transition and emission parameters from the 19F model fitted with the correct observations when reconstructing the most likely route taken through the hidden states. Results were inspected to ensure this did not cause systematic overestimation when compared with previous studies.

We found that the fit for NT swabs produced results which overestimated carriage duration when compared to previously reported estimates. The best fit to the model overestimated the $e_{21}$ parameter, which measures the false negative rate of swabbing, in favour of reduced transition intensities. We therefore fitted the model again, fixing this rate at 0.12. We based this figure on non-typable *Streptococcus pneumoniae* abundance as defined by 16S survey sequencing. At 1% proportional abundance in the sample, 12% came out as culture negative (Table 1).

**Table 1.** Success of culturing unencapsulated *S. pneumoniae*. Based on having >1% abundance of 16S reads showing the bacteria as being present, 44/361 true positive swabs were not successfully cultured.

Abundance	Culture positive	Number
>1%	Cultured	361
>1%	Not cultured	44
<1%	Cultured	56
<1%	Not cultured	54

**Figure 3.**
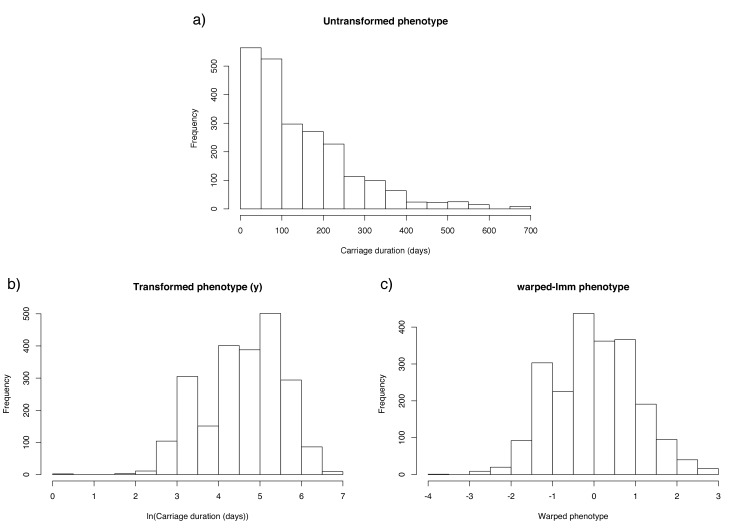
Distribution of carriage duration, and effect of monotonic transformation. Panel (**a**) shows a histogram of the inferred carriage duration, (**b**) shows this result after the natural logarithm is taken, and (**c**) after the warping function is applied. 10.7554/eLife.26255.010Figure 3—source data 1.Sequenced isolates and their untransformed inferred carriage durations. 10.7554/eLife.26255.011Figure 3—source data 2.Sequenced isolates and their warped carriage durations.

From all the swab data, we estimated that there were a total of 4382 carriage episodes (7.3 per child), of which 2254 had a complete set of AMR data available (Figure 3). After removing ten outlier observations from swabs taken accidentally during disease, we were able to match 2157 sequenced genomes with a carriage duration. Duration was positively skewed due to some observations of very long carriage times. We therefore took a monotonic transform of the carriage duration using warped-lmm to maximise the study's power to discover associations and estimate heritability (Figure 3). This uses a sum over three nonlinear step functions, plus a linear term, to transform the residuals into Gaussians (Snelson et al., 2004).

**Figure 3—figure supplement 1.**
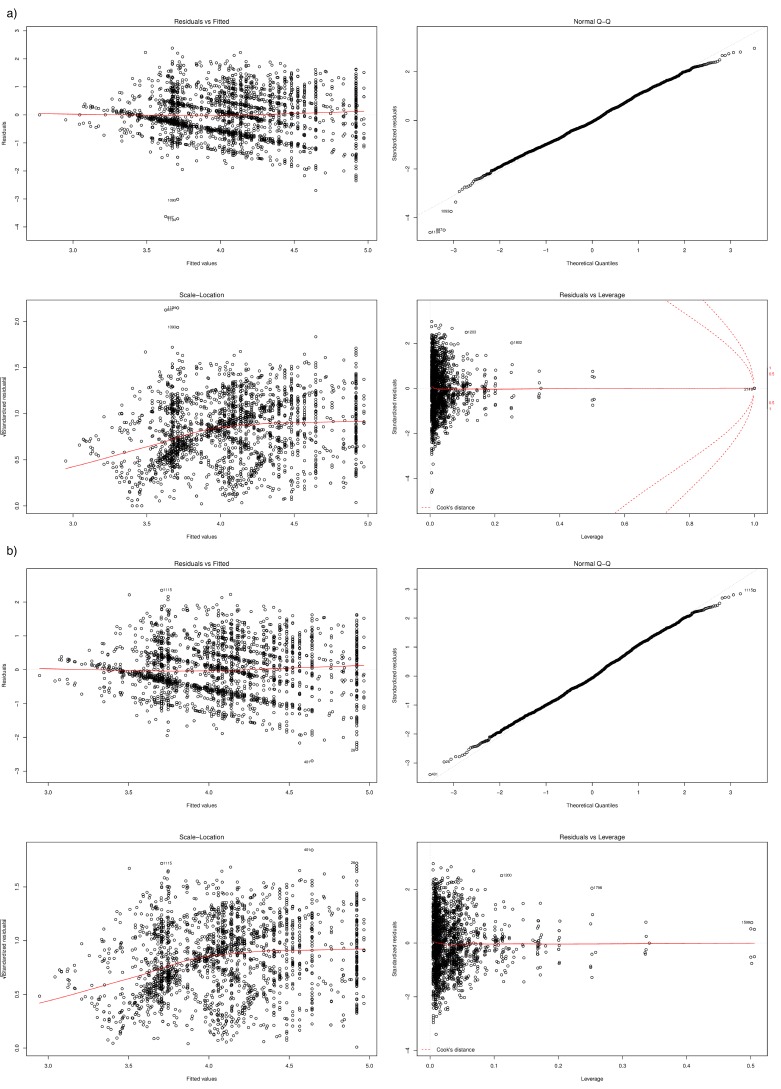
Regression diagnostics and outlier removal. Panel (a) shows prior to outlier removal, (b) after outlier removal as produced by plot.lm() in R. Points deviating from normal residuals (top right plot), and at high leverage (bottom right plot) were removed. These observations appeared to be due to swabs not taken at the prescribed monthly intervals.

**Figure 3—figure supplement 2.**
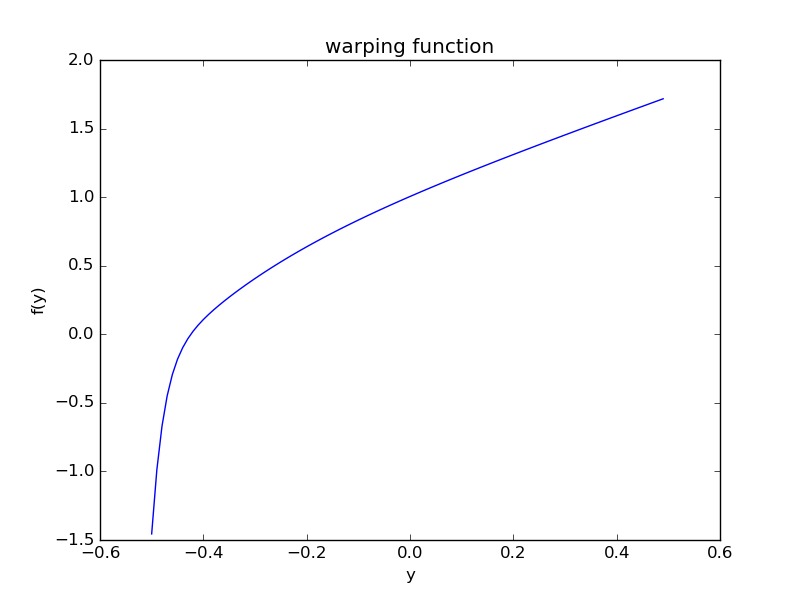
Monotonic warping function from warped-lmm. x-axis shows the centred and normalised input phenotype; y-axis shows corresponding warped value.

**Figure 3—figure supplement 3.**
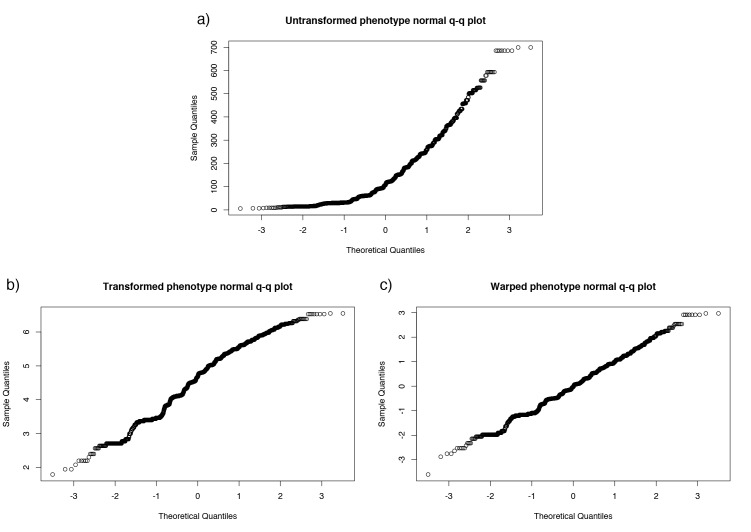
Normal quantile-quantile plot of carriage length, and effect of monotonic transformation. Panel (a) the inferred carriage duration, (b) after the natural logarithm is taken, and (c) after the warping function is applied.

### Overall heritability of carriage duration is high

The variation in carriage duration $\sigma_{P}^{2}$ is partly caused by variance in pneumococcal genetics, and variance in other potentially unknown factors such as host age and host genetics. It is common to write this sum as two components: genetic effects $\sigma_{G}^{2}$ and environmental effects $\sigma_{E}^{2}$. The proportion of the overall variation which can be explained by the genetics of the bacterium is known as the broad-sense heritability $H^{2}=\frac{\sigma_{G}^{2}}{\sigma_{G}^{2}+\sigma_{E}^{2}}$. Variants which are directly associated with carriage duration independently of other variants (non-epistatic effects) contribute to the narrow-sense heritability $h^{2}$, which is smaller than the overall broad-sense heritability (Visscher et al., 2008).

**Figure 4.**
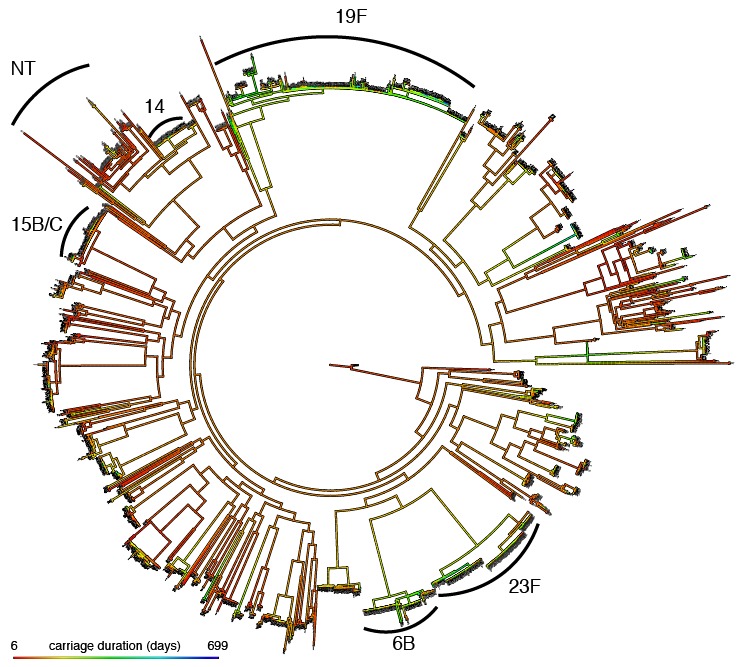
Mapping of carriage duration onto phylogeny. Using the carriage duration as a continuous trait, the ancestral state at every node of the rooted phylogeny was reconstructed. Red branches are carriage for a short time, blue for a long time. Clusters identified in previous analysis have been labelled. 10.7554/eLife.26255.017Figure 4—source data 1.Phylogenetic tree in Newick format.

$H^{2}$ can be estimated by linear regression on the phenotype of donor-recipient pairs which nearly share their genetics (Fraser et al., 2014). However in this dataset we were only able to confidently identify five transmission events, which was not enough to apply this method. Alternatively, analysis of variance of the phenotype between pathogens with similar genetics can be used to estimate heritability (Anderson et al., 2010). By applying this to phylogenetically similar bacteria (Figure 4), we estimated that $H^{2}=0.634$ (95% CI 0.592–0.686). This implies that the genetics of *S. pneumoniae* is an important factor in determining carriage duration in this population. If environmental conditions are associated with streptococcal genotype between populations (such as host vaccination status) the heritability estimate may differ.

**Figure 4—figure supplement 1.**
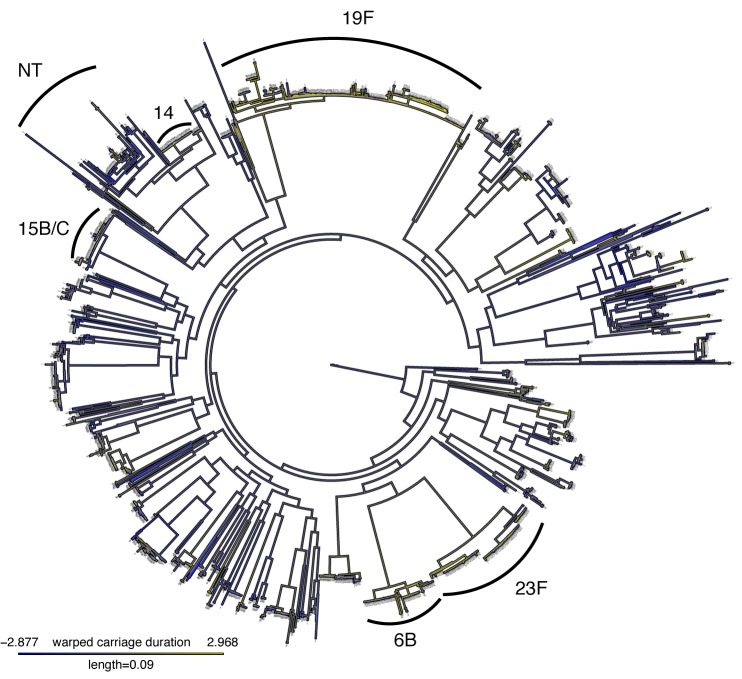
Mapping of warped carriage duration onto phylogeny. As Figure 4, but using the warped carriage duration. Blue branches are carriage for a short time, yellow for a long time.

**Figure 4—figure supplement 2.**
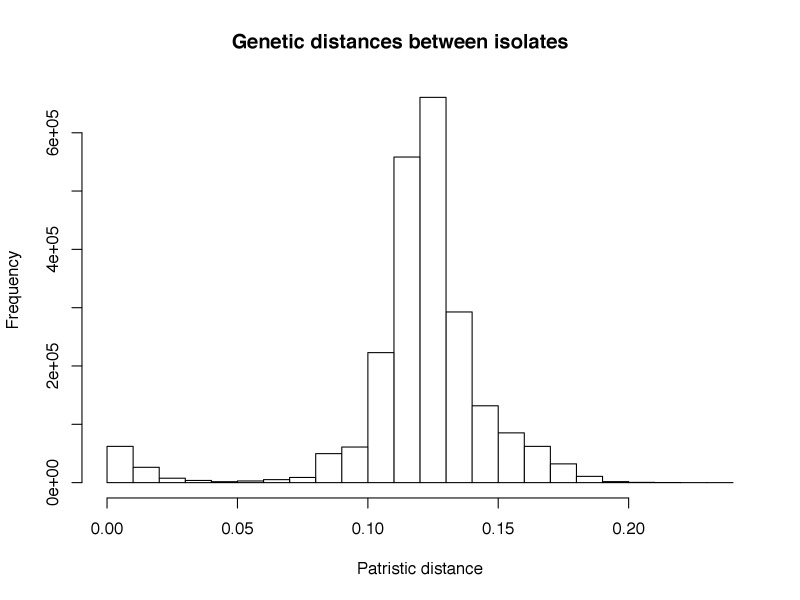
Histogram of pairwise patristic distances on the inferred phylogeny. A cut-off for heritability estimation was chosen at 0.04, under which a clear second maxima corresponds to closely related isolates on the tree.

**Figure 4—figure supplement 3.**
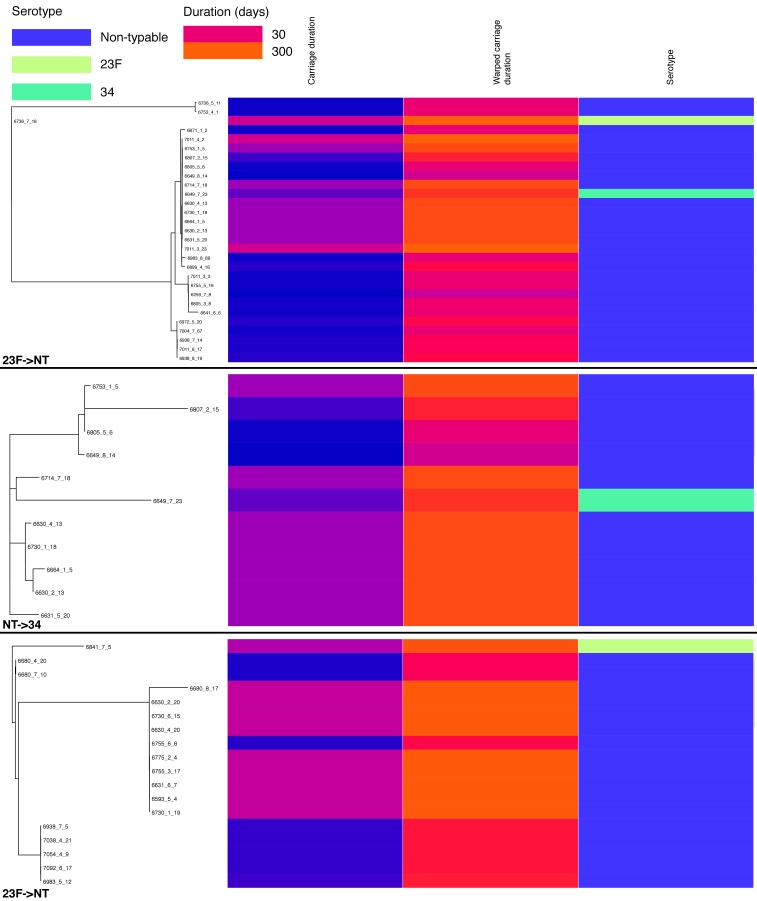
Change in carriage duration associated with capsule switching events. For each of the three events analysed the subtree containing the switch is shown on the left. For each isolate within the subtree, carriage duration (on a roughly exponential scale), warped carriage duration (on a roughly linear scale) and serotype are shown as coloured bars aligned with the tip.

**Figure 4—figure supplement 4.**
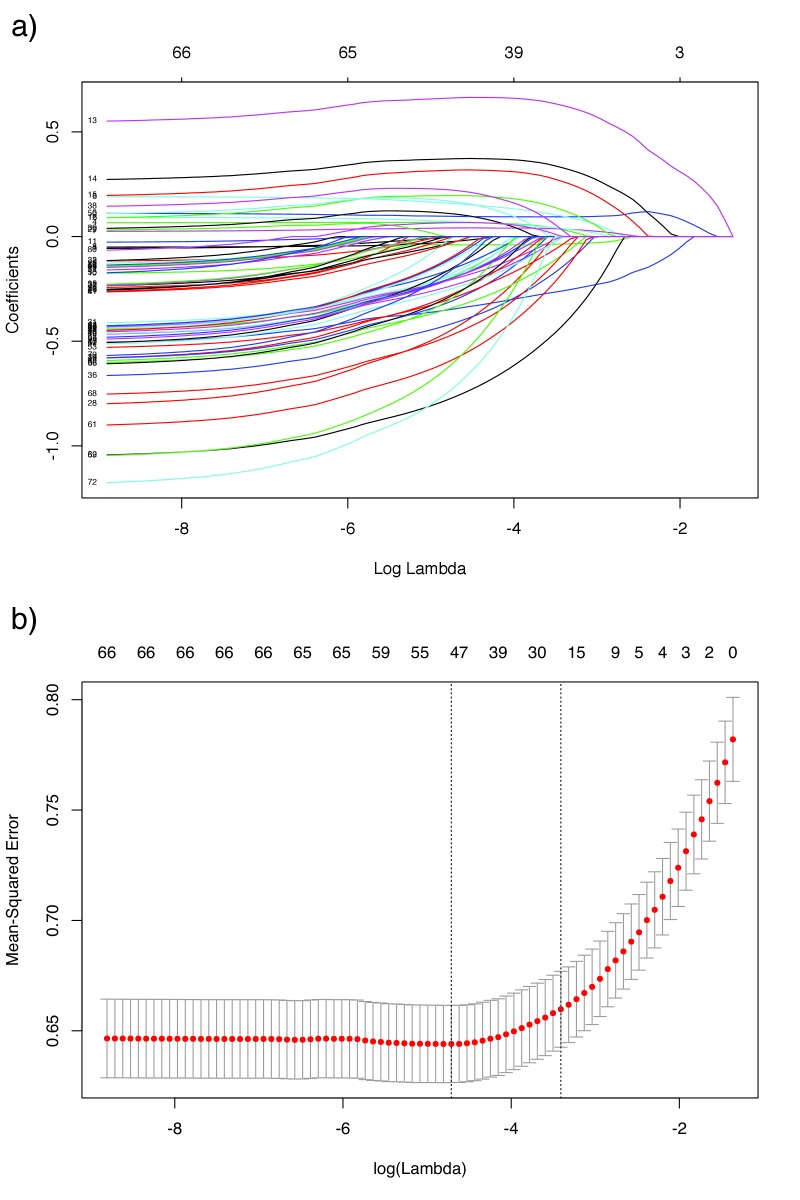
Lasso regression plots for lineage effects. Panel a) shows the value of each predictor on the y-axis for different values of the $\ell_{1}$ penalty λ on the x-axis, which increases from left to right. The labels along the top are the number of predictors remaining in the model for each λ. Panel b) shows the results of leave-one-out cross validation on the mean-squared error, along the same x-scale. The λ at minimum error is shown by the left dashed line, and the λ within one standard error is shown by the right dashed line.

A lower bound on $h^{2}$ can be calculated by fitting a linear mixed model through maximum likelihood to common SNPs ($h_{\mathrm{SNP}}^{2}$) (Lee et al., 2011; Manolio et al., 2009). We used the model in warped-lmm (Fusi et al., 2014) to estimate $h_{\mathrm{SNP}}^{2}$ for carriage duration data, yielding an estimate of 0.445, consistent with our estimate for $H^{2}$.

### Serotype and drug resistance explain part of the narrow-sense heritability

After calculating the overall heritability, we wished to determine the amount that the specific variation in the pathogen genome contributes to changing carriage duration. In the context of genome wide association studies (GWAS) in bacteria strong linkage-disequilibrium (LD) is present across the entire genome, making it difficult to pinpoint variants associated with carriage duration and not just present in the background of longer or shorter carried lineages (Chen and Shapiro, 2015). In *S. pneumoniae*, serotype and antibiogram are correlated with the overall genome sequence (Brueggemann et al., 2003; Chewapreecha et al., 2014a; Enright and Spratt, 1998). If these factors are associated with carriage duration, large sets of variants which define long-carried and short-carried lineages will be correlated with carriage duration in a naive association test (Chen and Shapiro, 2015; Read and Massey, 2014).

A distinction has therefore been made between variants which evolve convergently and affect a phenotype independently of lineage – termed locus effects – to those which are collinear with a genotype which is associated with the phenotype, termed lineage effects (Earle et al., 2016). Locus effects may be associated with a change in carriage duration due to convergent evolution (which may occur through recombination between lineages). In such regions, the causal loci and corresponding phenotypic effects are easier to identify (Power et al., 2017). Linear mixed models can be used to find these variants which are associated with a bacterial phenotype independent of lineage; discovery of homoplasic and polygenic variation associated with the phenotype across the entire tree is well powered (Earle et al., 2016).

While the high heritability suggests many pathogen variants do affect carriage duration, it does not give information on how many of these will be locus or lineage effects. We mapped carriage duration onto the phylogeny, reconstructing the ancestral state at each node. Consistent with the high heritability of carriage duration we found that carriage length was clearly stratified by lineage (Figure 4): we calculated Pagel’s lambda as 0.56 ($p<10^{-10}$). We also modelled the evolution of carriage duration along the tree using an Ornstein-Uhlenbeck model, and found that lineage genetics was significantly correlated with the trait (LRT = 952; $p<10^{-10}$)

We first tested for the association of serotype with carriage duration using lasso regression and with a linear-mixed model (LMM). Serotype is correlated with sequence type (Croucher et al., 2011) and has previously been associated with differences in carriage duration (Abdullahi et al., 2012a; Turner et al., 2012). We also included resistance to six antibiotics, the causal element to some of which are known to be associated with specific lineages (Lees et al., 2016). These are therefore possible lineage effects which would be unlikely to be found associated under a model which adjusts for population structure (Chen and Shapiro, 2015).

Not all serotypes and resistances may have an effect on carriage duration, or there may not be enough carriage episodes observed to reach significance. As including extra predictors in a linear regression always increases the variance explained, we first performed variable selection using lasso regression (Efron et al., 2004) to obtain a more reliable estimate of the amount of variation explained. Where a resistance and serotype are correlated and both associated with a change in carriage duration, this will produce a robust selection of the predictors (Hebiri and Lederer, 2012).

The selected predictors and their effect on carriage duration are shown in Table 2. The total variance explained by these lineage factors was 0.19, 0.178 for serotype alone and 0.092 for resistance alone. When we used genomic partitioning of variance components these were instead estimated to be 0.253, 0.135 and 0.113, respectively. We applied the covariance test (Lockhart et al., 2014) to determine which lineage effects were significantly associated with carriage duration and found that 19F, erythromycin resistance, 23F, 6B caused significant (α<0.05) increase in carriage duration and being non-typable caused a significant decrease.

**Table 2.** Coefficients from lasso regression model of carriage duration. The mean (intercept) corresponds to a sensitive 6A/C carriage episode, and different serotypes and resistances are perturbations about this mean. Positive effects are expected to have a greater magnitude, due to the positive skew of carriage duration. Rows in bold were significant predictors in the covariance test.

Factor	Effect on carriage duration (days)
**Mean (intercept)**	**59.5**
**Erythromycin resistance**	+7.5
Tetracycline resistance	+3.0
Trimethoprim resistance	+2.9
Clindamycin resistance	+1.8
Penicillin intermediate resistance	+1.3
**Serotype 19F**	**+46.9**
**Serotype 23F**	**+21.0**
**Serotype 6B**	**+16.2**
Serotype 14	+7.2
Serotype 21	+1.6
Serotype 19B	−0.1
Serotype 18C	−1.9
Serotype 29	−4.3
Serotype 3	−4.5
Serotype 4	−7.2
Serotype 24F	−8.5
**Non-typable (NT)**	**−12.3**
Serotype 5	−18.6

Previous studies have used isogenic strains to look for effects of serotype of colonisation and carriage duration independent of genetic background. Resistance to killing (Weinberger et al., 2009), growth phenotype (Hathaway et al., 2012) and resistance to complement (Melin et al., 2010) have all been shown to affect carriage through serotype rather than genetic background. Conversely, some bacterial genetic variation has been shown to be able to affect colonisation independent of serotype (Khan et al., 2014).

We therefore wished to test whether the detected effect of serotype and resistance on carriage duration was entirely mediated through their covariance with lineage, or whether they are independently associated with carriage duration. We first looked for differences in duration over three recent capsule gain/loss events; if there is an effect of serotype independent of genetic background, these would be predicted have the largest difference between serotypes while controlling for the relatedness of isolates. No significant difference in duration was seen between isolates with or without capsule within the same lineage (p=0.39; Figure 4).

However, as these events were limited in number, assumed genetic independence within the clade and occurred only in part of the population, we also performed the same regression as above while also including lineage (defined by discrete population clusters) as a predictor. This therefore allows serotypes which appear in different population clusters to distinguish whether lineage or serotype had a greater effect on carriage duration. The covariance test found that 19F, erythromycin resistance and being non-typable had significant effects on the model (in that order). As these terms enter the model before any lineage specific effect, this suggested these serotypes and resistances are associated with variation in carriage duration independent of background genotype

This lasso-based analysis may be vulnerable to confounding from unmeasured variables which may be associated with the explanatory variables (serotype and resistance). To fully account for the effect of the bacterial genome rather than relying on discrete clusters as covariates in the regression, we performed regression of these lineage effects under an LMM where the relatedness between strains was instead included as a random effect. The predictors had the same order of significance, but only serotype 19F reached genome-wide significance ($p=3.8\times 10^{-7}$).

Together, this suggests that the main lineage effect on carriage duration is the serotype, but only some serotypes (19F) have an association independent of genetic background. We also found that erythromycin resistance may be significantly associated with an increased carriage duration. While being a relatively uncommon treatment in this setting (3% of treatments captured), we did not find that other antibiotics were associated. This may be because erythromycin resistance would be expected to cause an almost four order magnitude increase in minimum inhibitory concentration (MIC), whereas other resistance acquisitions have a much smaller effect.

Additionally, we calculated the mean sojourn times (average length of time children are expected to remain in the carrying state of the model with the given serotype) and mean number of carriage episodes from the fit to the HMM for commonly carried serotypes (), which gave results similar to the regression performed above. These estimates are comparable to the previous analysis on a subset of these samples. The majority of carriage episodes were due to five of the seven paediatric serotypes (Shapiro and Austrian, 1994), or non-typeable isolates. The results show 19F, 23F and 14 were carried the longest, 6A/C and 6B for intermediate lengths, and NT the shortest.

**Table 3.** Mean length of carriage, and expected number of carriage episodes within the first two years of life. Only serotypes with enough data for the HMM fit to converge are shown. Starred observations have a standard error which is larger than the estimated value, indicating low confidence in the estimate.

Serotype	Sojourn time (days)	Expected number of infections
19F	292*	0.85
23F	112	0.83
6A/C	76.4	0.88
6B	114	0.75
14	137*	0.58
NT	40.6	2.05

The overall picture of the first two years of infant carriage is one containing one or two long (over 90 day) carriage episodes of a common serotype (6A/C, 6B, 14, 19F, 23F) and around two short (under a month) carriage episodes of non-typable *S. pneumoniae*. Colonisation by other serotypes seem to cause slightly shorter carriage episodes, though the relative rarity of these events naturally limits the confidence in this inference. That some serotypes are rarer and carried for shorter time periods may be evidence of competitive exclusion (Hardin, 1960; Trzciński et al., 2015), as fitter serotypes quickly replace less fit serotypes thus leading to reduced carriage duration. The calculated mean carriage duration of NT pneumococci is similar to the minimum resolution we were able to measure by the study design, which suggests carriage episodes may actually be shorter than one month. Unfortunately the only existing study with higher resolution did not check for colonisation by NT pneumococci (Abdullahi et al., 2012a).

These estimates are similar to previous longitudinal studies in different populations (Hill et al., 2010; Högberg et al., 2007; Melegaro et al., 2007), though against the Kilifi study our estimates are systematically larger. This may be due to the lower resolution swabbing we performed, or may be because the previous study was unable to resolve multiple carriage (11% of positive swabs). While our heritability estimates are specific to this population due to differences in host, vaccine deployment and transmission dynamics, the similarity of the estimates of serotype effect to those from different study populations suggests our results may be somewhat generalisable.

### Additional loci identified by genome-wide association

**Figure 5.**
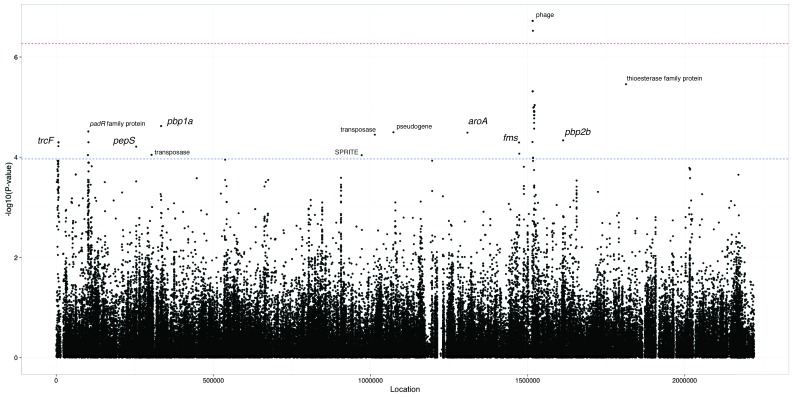
Manhattan plot of SNPs associated with carriage duration. The significance of each SNP’s association with carriage duration against its position in the ATCC 700669 genome is shown. The red line denotes genome-wide significance (α<0.05 Bonferroni corrected with 92487 unique tests), and the blue line suggestive significance (2.3 orders of magnitude below significant, following convention). Loci reaching suggestive significance are labelled with their nearest annotation, as in Table 4. 10.7554/eLife.26255.026Figure 5—source data 1.Plot file for Manhattan plot, with coordinates and -log10 transformed p-values of all tested SNPs.

To search for locus effects as discussed above, we applied an LMM to all the common SNPs and k-mers in the dataset. The results for SNPs are shown in Figure 5 and Table 4, with 14 loci reaching suggestive significance and two reaching genome-wide significance (top hit β=0.17; $p=2.1\times 10^{-7}$; MAF = 1%). We also found that 424 k-mers reached genome-wide significance (top hit β=0.11; $p=2.1\times 10^{-12}$; MAF = 2%), which we filtered to 321 k-mers over 20 bases long to remove low specificity sequences (Figure 5). To determine their function, we mapped these k-mers to the coordinates of reference sequences.

**Figure 5—figure supplement 1.**
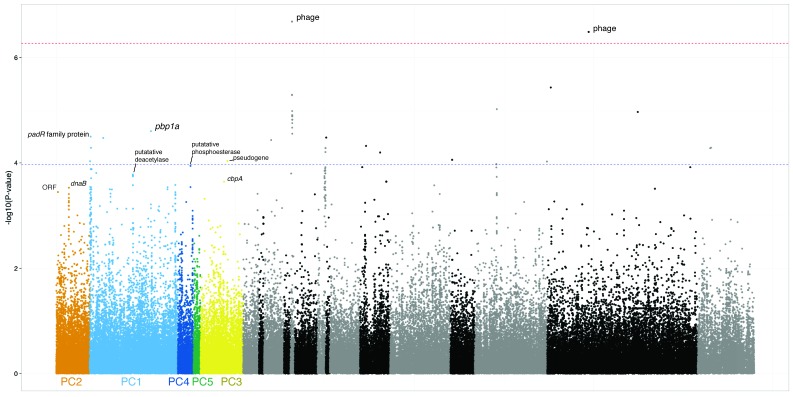
Possible SNPs associated with lineage and carriage duration. The SNPs and p-values as shown in Figure 5, however the x-axis is now ordered by strength of association of lineage (defined by principal component) with carriage duration. The left most lineages are those most associated, those in black/grey were not significantly associated. SNPs are coloured by the lineage they are most associated with.

**Figure 5—figure supplement 2.**
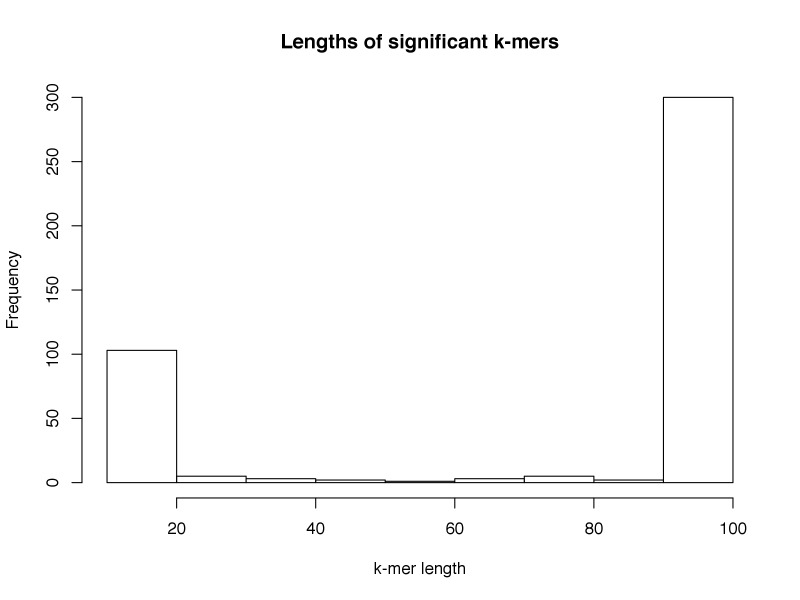
Distribution of lengths of significant k-mers. The lengths of those k-mers reaching significance in the LMM analysis. Lengths below 20 bases were filtered from downstream analysis, due to having low specificity.

**Figure 5—figure supplement 3.**
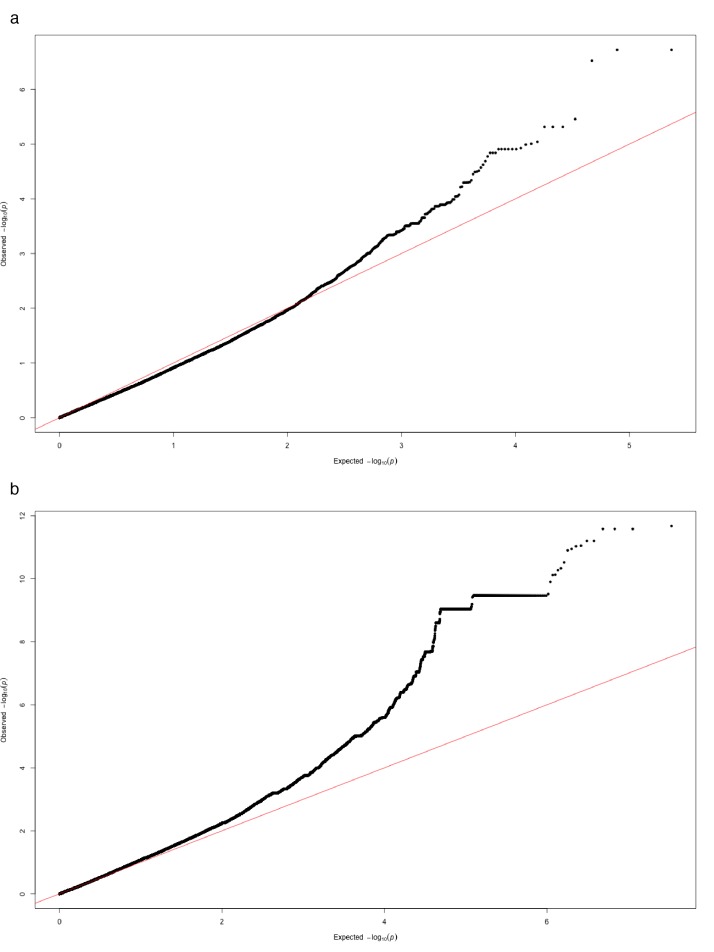
Quantile-quantile plot of association p-values. For fast-lmm results on (a) SNPs passing quality filters and (b) k-mers of all lengths passing quality filters.

**Figure 5—figure supplement 4.**
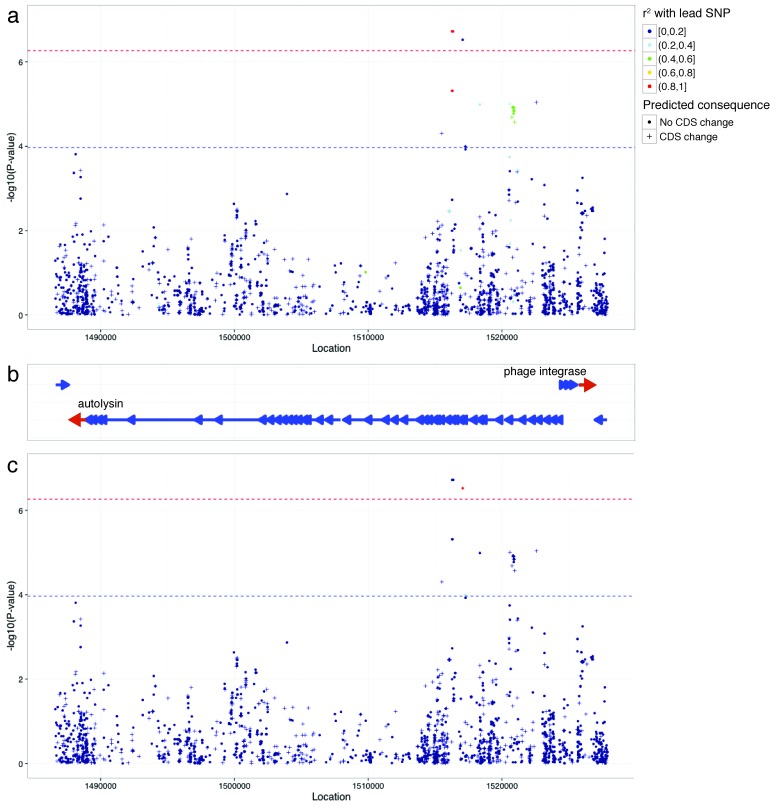
Manhattan plots of phage-associated SNPs associated with carriage duration. As in Figure 5, but enlarging the phage region found to be significant. SNPs are coloured by their LD with the lead SNP (the highest P-value in the region plotted), and are crosses if they are predicted to cause a change in coding sequence. Panel (a) shows LD in relation to the lead SNP at position 1516350. Panel (b) plots genes in the region, with the start and end of the phage genes labelled. Panel (c) shows LD in relation to the second SNP signal at position 1517063.

**Figure 5—figure supplement 5.**
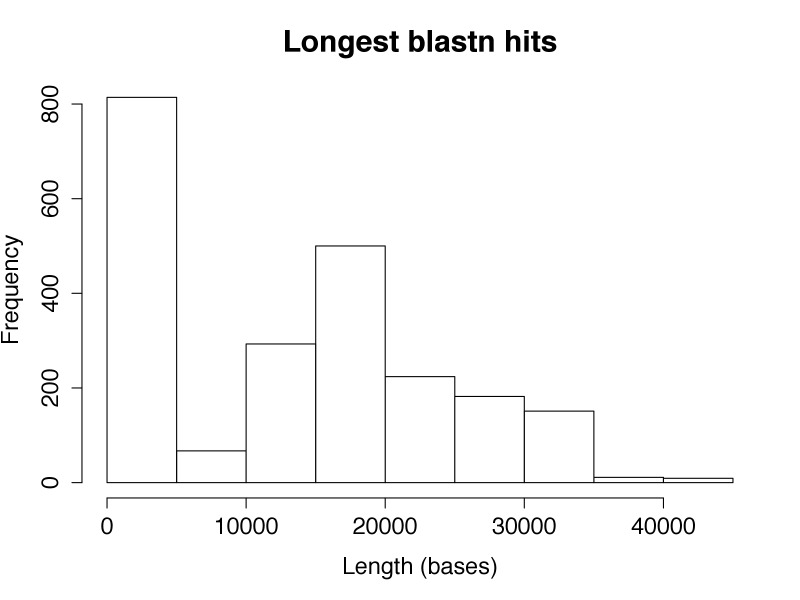
Identification of phage in assemblies by blastn hit length. Histogram of the length of top hits against a database of phage sequence by blastn. Isolates with >5000 bp hits were defined as having phage present.

**Table 4.** SNP Locus effects at genome-wide and suggestive significance. Co-ordinates are with respect to the ATCC 700669 reference genome, and are for the lead SNP in each locus after LD-pruning. Effect sizes are for the warped phenotype. 10.7554/eLife.26255.028Table 4–source data 1.Association results for SNPs, from fast-lmm.

Co-ordinate	Nearest annotation	Effect size	P-value	Significance level
6753	*trcF*	−0.12	$6.2\times 10^{-5}$	Suggestive
254312	*pepS*	−0.11	$6.4\times 10^{-5}$	Suggestive
303239	IS630-Spn1 transposase	0.078	$9.2\times 10^{-5}$	Suggestive
333632	*pbp1a*	0.079	$2.5\times 10^{-5}$	Suggestive
971849	SPRITE repeat region	0.078	$9.4\times 10^{-5}$	Suggestive
1013978	IS630-Spn1 transposase	0.11	$3.7\times 10^{-5}$	Suggestive
1073185	FM211187.3435 (pseudogene)	0.086	$3.3\times 10^{-5}$	Suggestive
1308604	*aroA*	−0.27	$3.8\times 10^{-5}$	Suggestive
1472933	Upstream of *fms*	−0.23	$5.3\times 10^{-5}$	Suggestive
1473700	putative glutathione S-transferase	−0.16	$8.8\times 10^{-5}$	Suggestive
1515497	hypothetical phage protein	−0.099	$5.2\times 10^{-5}$	Suggestive
1516293	putative phage Holliday junction resolvase	−0.10	$5.1\times 10^{-6}$	Suggestive
1516350	putative phage Holliday junction resolvase	−0.12	$2.1\times 10^{-7}$	Genome-wide significant
1517063	phage protein	−0.11	$3.3\times 10^{-7}$	Genome-wide significant
1613197	*pbp2b*	−0.21	$4.8\times 10^{-5}$	Suggestive
1813192	thioesterase superfamily protein	−0.12	$4.8\times 10^{-6}$	Suggestive

The only genome-wide significant SNP hits are synonymous changes (MAF = 1%) in the replication module of the prophage in the ATCC 700669 genome (Croucher et al., 2009), a highly variable component of the pneumococcal genome (Croucher et al., 2014a) (Figure 5). The LD structure suggested there were two separate significant signals found in this region. We therefore performed another GWAS conditioning on the top hit to test if there was a second independent signal, but found that the second hit in this region was no longer significant (position 1526024; $p=2.2\times 10^{-4}$). The current data is therefore consistent with only a single significant hit to prophage.

The most significant k-mer hits were also located in phage sequence (MAF 2%) and were associated with a reduced duration of carriage. As these mobile genetic elements are less weakly population stratified than other regions of the genome, they are easier to find as locus effects. The LD in this region is less than in the rest of the genome, as prophage sequence is highly variable within *S. pneumoniae* lineages (Croucher et al., 2014a). Multiple independent phage variants may therefore affect carriage duration, which will increase their significance using a LMM. Indeed, the significant results from the LMM (top SNP $p=2.1\times 10^{-7}$; top k-mer $p=2.1\times 10^{-12}$) are not significant (top SNP $p=5.1\times 10^{-6}$; top k-mer $p=5.7\times 10^{-8}$) under a model of association using a linear regression with the first 30 principal components as fixed effects to control for population structure rather than random effects, and are strongly associated with the population structure components of the model (highest association $p=5.2\times 10^{-75}$ with PC 2).

We postulated that presence of any phage in the genome may cause a reduction in carriage duration. By using the presence of phage as a trait under the same linear mixed model, we however found no evidence of association when correcting for population structure (p=0.35). These results are therefore evidence that infection with a specific phage sequence is associated with a slight decrease in carriage duration. A similar result has previously been found in a genome-wide screen in *Neisseria meningitidis*, where a specific phage sequence was found to affect the virulence and epidemiology of strains (Bille et al., 2005; Bille et al., 2008). Additionally, previous in vivo tests have shown phage elements to cause a fitness decrease of *S. pneumoniae* during carriage (DeBardeleben et al., 2014).

The genetic polymorphisms in the prophage associated with changes in carriage duration, found in 2% of viral sequences, are found within coding sequences inside the phage replication module (Romero et al., 2009). It is unlikely the specific variants of these proteins cause a significant difference in cell phenotype, because they are only highly expressed after the prophage is activated, and cell lysis is typically imminent. One explanation for these results is that a subpopulation of prophage do not cause a significant decrease in their host bacterium's carriage duration, which could be due to beneficial ‘cargo’ genes. Yet previous surveys of pneumococcal prophage have found little evidence of these elements carrying such sequences (Croucher et al., 2014a; Romero et al., 2009). One phage protein that has been found to alter the bacterial phenotype is PblB, a phage structural protein that can also mediate bacterial adhesion to human cells (Loeffler and Fischetti, 2006). However, *pblB* is within the morphology module (Romero et al., 2009) and as an adhesin might be expected to increase carriage duration. Hence the detected association is unlikely to represent expression of viral machinery or cargo genes in the host cell while the prophage is dormant.

Alternatively, the association with only a subset of prophage may be the consequence of sampling. Using a monthly swabbing approach, it was only possible to robustly infer changes in the carriage duration of genotypes that colonise hosts for long periods. Therefore any prophage locus that enhances a virus’ ability to infect long carriage duration pneumococci may have an elevated correlation with the variation in the observed phenotype. As phage commonly exhibit high levels of strain specificity (Duplessis and Moineau, 2001), this is a plausible mechanism, although the role of the replication module in such host preference is unclear.

An additional mechanism by which prophage can affect host phenotype is by inserting into, and thereby disrupting, functional genes. Pneumococcal prophage frequently insert into *comYC*, thereby preventing the host cell undergoing transformation (Croucher et al., 2011; Croucher et al., 2014b). Using previous categorisation of the *comYC* gene in this collection into intact versus interrupted or missing (Croucher et al., 2016), we found that having an intact *comYC* gene (23% of isolates) was significantly associated with an increased carriage duration using the LMM (β=0.29; $p=1.4\times 10^{-44}$). The effect size is similar to the associated phage k-mers, but has at a higher allele frequency (hence the increased significance of the result). An interpretation consistent with these findings would be that the effect of phage k-mers is actually through interrupting *comYC*. The k-mers themselves were spread out to lower frequencies due to their sequence variability, and no references used allowed mapping to find the *comYC* interruption directly.

Signals at the suggestive level include *pbp1a* and *pbp2b*, which suggest as above that penicillin resistance may slightly increase carriage duration, but there are not enough samples in this analysis to confirm or refute this. Other signals near genes at a suggestive level included SNPs in *trcF* (transcription coupled DNA repair), *padR* (repressor of phenolic acid stress response), *pepS* (aminopeptidase), *aroA* (aromatic amino acid synthesis), *fms* (peptide deformylase) and a thioesterase superfamily protein. K-mers from erythromycin resistance genes (*ermB*, *mel*, *mef*) were expected to reach significance from the above analysis, but did not: it has however previously been shown that the power to detect these elements in a larger sample set taken from the same population is limited due to the multiple resistance mechanisms and stratification of resistance with lineage (Lees et al., 2016).

The test statistic from fast-lmm roughly followed the null-hypothesis, with the exception of the significant phage k-mers (Figure 5). However there is limited power to detect effects associated with both the lineage and phenotype. This effect has been previously noted, and while LMMs have improved power for detecting locus specific effects they lose power when detecting associated variants which segregate with background genotype (Earle et al., 2016).

To search for candidate regions which may be independently associated with both a lineage and increased carriage duration, we ran an association test using a set number of fixed effects as the population structure correction. This is expected to have higher power than an LMM for true associated variants on ancestral branches, but will also increase the number of false positives (variants co-occurring on these branches which do not directly affect the carriage duration themselves). We also tested SNPs for their association with those principal components which were themselves significantly associated with carriage duration, and therefore may be driving the lineage associations (Earle et al., 2016).

The most highly associated SNPs were in all three *pbp* regions associated with β-lactam resistance, the capsule locus, *recA* (DNA repair and homologous recombination), *bgaA* (beta-galactosidase), *phoH*-like protein (phosphate starvation-inducible protein), *ftsZ* (cell division protein) and *groEL* (chaperonin). As 19F, the serotype most associated with carriage duration, is predominantly the β-lactam resistant PMEN14 lineage the *pbp* association may be driven through strong LD between with this serotype. Figure 5—figure supplement 1 shows the analysis of SNPs which may be driving significant lineage associations – this also suggested *dnaB* (DNA replication) may be associated with altered carriage duration. Associated k-mers were also found in *phtD* (host cell surface adhesion), *mraY* (cell wall biosynthesis), *tlyA* (rRNA methylase), *zinT* (zinc recruitment), *adcA* (zinc recruitment) and *recJ* (DNA repair). Additionally we found k-mers in the bacteriocin *blpZ* and immunity protein *pncM* (Bogaardt et al., 2015) to be associated with variability in carriage duration. This could be evidence that intra-strain competition occurs within host via this mechanism, consistent with previous in vitro mouse models (Dawid et al., 2007).

It is not possible to determine whether variation in these genes is associated with a change in carriage duration or if the variation is present in longer carried, generally more prevalent lineages. For example, β-lactam resistance may appear associated as the long carried lineages 19F and 23F are more frequently resistant, or it may genuinely provide an advantage in the nasopharynx that extends carriage duration independent of other factors. Future studies of carriage duration, or further experimental evidence will be needed to determine which is the case for these regions.

Antigenic variation in known regions (of *pspA*, *pspC*, *zmpA* or *zmpB*) may be expected to cause a change in carriage duration (Lipsitch and O'Hagan, 2007), however we found none of these to be associated with a change in carriage duration. This was likely due to stratification of variation in these regions with lineage, but may also be caused by a larger diversity of k-mers in the region reducing power to detect an association.

### Child age independently affects variance in carriage duration

Finally, we wished to determine the importance of two environmental factors which are known to contribute to variance in this phenotype: child age and whether the carriage episode is the first the child has been exposed to (Abdullahi et al., 2012a; Abdullahi et al., 2012b; Turner et al., 2012). These have been applied throughout the analysis as covariates, both in the estimation of carriage episodes and in associating genetic variation with change in carriage duration.

**Figure 6.**
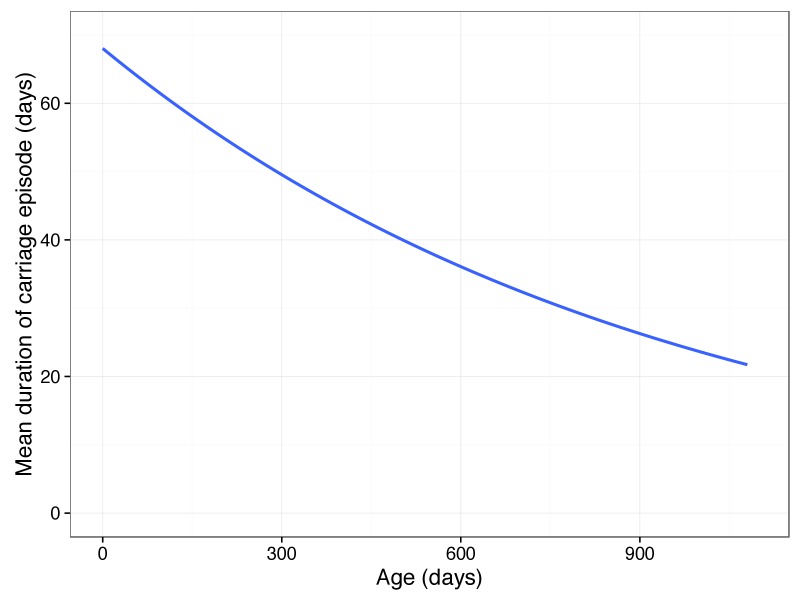
Predicted mean carriage duration as a function of child age. Fit is an exponential decay over the first two years of life, using the decay rate inferred from a linear regression of log(carriage duration).

We applied linear regression to these factors while using the first 30 PCs to correct for the effect of the bacterial genome, which showed they were both significantly associated with carriage duration as expected (age $p=3.9\times 10^{-7}$; previous carriage $p=2.5\times 10^{-8}$). Using the linear mixed model to control for bacterial genotype both factors were again significant (LRT = 26.4; $p=1.8\times 10^{-6}$). Together, they explained 0.046 of variation in carriage duration. As found previously, increasing child age contributes to a decrease in the duration of carriage episodes. From a mean of 68 days long, we calculated a drop of 19 days after a year, and 32 days after two years. Extrapolating, this causes carriage episodes longer than two days to cease by age 11 (Figure 6). Previous carriage of any serotype was estimated to cause an increase in the duration of future carriage episodes, though previous studies have found no overall effect (Weinberger et al., 2008). It has previously been shown that prior exposure to non-typables in this cohort make colonisation by another non-typable occur later, and for a shorter time (Turner et al., 2012). The positive effect observed in this analysis is therefore likely to be an artefact due to subsequent carriage episodes being more likely to be due to typable pneumococci.

Additional environmental factors that explain some of the remainder of the variance may include the variation of the host immune response and interaction with other infections or co-colonisation. In particular, co-infection influenza A was not recorded but is known to affect population dynamics within the nasopharynx (Kono et al., 2016). Fundamentally, imprecise inference of the carriage duration will limit our ability to fully explain its variance.

## Discussion

Other than serotype, the genetic determinants of pneumococcal carriage duration were previously unknown. By developing models for longitudinal swab data and combining the results with whole genome sequence data we have quantified and mapped the genetic contribution to the carriage duration of *S. pneumoniae*. We found that despite a range of other factors such as host age which are known to cause carriage duration to differ, sequence variation of the pneumococcal genome explains most of this variability (63%). Common serotypes and resistance to erythromycin cause some of this effect (19% total), as does the presence or absence of particular prophage sequence in the genome. Table 5 summarise the sources we found to be significantly associated with variation in carriage duration.

**Table 5.** Summary of variance of carriage duration explained by genetic and environmental factors. $H^{2}$ encompasses all rows, other than the measured environmental effects. For each variant component the method used to estimate it is reported: CPP - closest phylogenetic pairs; LMM - variance component using a linear mixed model with pathogen genotype as random effects; $R^{2}$ - linear regression using lasso to select predictors.

Source	Of which is	Total variance explained	Proportion of total heritability explained
Total heritability ($H^{2}$)		0.634 (CPP)	1.00
	Common SNP heritability ($h_{\mathrm{SNP}}^{2}$)	0.438 (LMM)	0.691
	Serotype and resistance	0.190 ($R^{2}$)/0.253 (LMM)	0.300 ($R^{2}$)/0.399 (LMM)
	Serotype only	0.178 ($R^{2}$)/0.135 (LMM)	0.281 ($R^{2}$)/0.213 (LMM)
	Resistance only	0.092 ($R^{2}$)/0.113 (LMM)	0.145 ($R^{2}$)/0.178 (LMM)
	Phage k-mers	0.067 (LMM)	0.106
	Intact *comYC*	0.127 (LMM)	0.201
Measured environmental effects	Age and previous carriage	0.046 ($R^{2}$)	-

We provide a quantitative estimate of how closely transmission pairs share their carriage duration, and show evidence for differences both between and within serotypes. The implication of phage as having a significant effect on carriage duration has interesting corollaries on pneumococcal genome diversification through frequent infection and loss of prophage, even during carriage episodes in this dataset.

Investigating a mechanism for the prophage association, we found that having an intact *comYC* gene, which is frequently interrupted by prophage causing loss of function of the competence system, was associated with increased carriage duration. While the competence system is observed to remain intact over the evolutionary history of the species, these disruptive mutations spread irreversibly through the population as competent bacteria can acquire the mutation, and non-competent bacteria can no longer reverse it through recombination (Croucher et al., 2014b). Selection must therefore maintain the function at this locus over short timescales, and an increased carriage duration may be evidence of this. We therefore hypothesise that the associated prophage sequences may affect carriage duration through disruption of the competence system, without which deleterious mutations will accumulate in the population due to Muller's ratchet.

The results presented here have important implications for the modelling of pneumococcal transmission and their response to perturbation of the population by vaccine. Importantly, our analysis of heritability shows that variants other than serotype affect carriage duration, consistent with recent theoretical work (Lehtinen et al., 2017). Here we have shown that these alleles do exist in a natural population, and also identified candidates for the loci which fulfil this role. Together these studies suggest that variants exist in the pneumococcal genome which alter carriage duration, which in turn is linked to antibiotic resistance.

We were not able to fully explain the basis for heritability of carriage duration for a number of reasons. The close association of the phenotype with lineage limited our power to fine-map lineage associated variants other than capsule type which may affect carriage duration. Meta-analysis with more large studies with higher resolution may help to resolve these issues: we are conducting a similar study in Cape Town, South Africa which will combine sequence data with two-weekly swabs and will be compared to these results in future. Additional environmental factors that explain some of the remainder of the variance may include the variation of the host immune response and interaction with other infections or co-colonisation. In particular, co-infection with influenza A was not recorded but is known to affect population dynamics within the nasopharynx (Kono et al., 2016).

This is a phenotype which would have been difficult to assay by traditional methods such as in an animal model due to the cohort size needed and the length of time experiments would need to be run for. By instead using genome-wide association study methods we have been able to quantitatively investigate a complex phenotype in a natural population. We believe that the analysis of heritability and variance explained in a phenotype of interest, as presented here, will be an important part analysis of complex bacterial traits in future studies.

## Materials and methods

### Sample collection

The study population was a subset of infants from the Maela longitudinal birth cohort (Turner et al., 2013a), and was split into two cohorts. In the ‘routine’ cohort, 364 infants were swabbed monthly from birth, 24 times in total. All swabs were cultured and serotyped using the latex sweep method (Turner et al., 2013b). In the ‘immunology’ cohort 234 infants were swabbed on the same time schedule, but cultured and serotyped following the World Health Organisation (WHO) method (Turner et al., 2012). Non-typable pneumococci were confirmed by bile solubility, optochin susceptibility and Omniserum Quellung negative. For both cohorts phenotypic drug resistance to six antibiotics was available (chloramphenicol, β-lactams, clindamycin, erythromycin, trimethoprim and tetracycline). 3161 randomly selected pneumococcal positive swabs from the study population have been previously sequenced, 2175 of which were from these longitudinal infant samples (Chewapreecha et al., 2014a).

### Converting swab data into a time series

Latex sweeps could not differentiate 6A and 6C serotypes, so we treated these as a single serotype when detected by this method (in WHO serotyping PCR was used to differentiate these serotypes). 15B and 15C serotypes spontaneously interconvert, so were combined. We removed two duplicated swabs (08B09098 from the immunology cohort; 09B02164 from the routine observation cohort).

To get a good fit of the HMM, we normalised observation times for each sample. Defining infant birth as t=0, subsequent sampling times $t_{i}$ were measured in days, and normalised to have a variance of one. The actual (untransformed) carriage duration in days was used as initial phenotype y.

### Hidden markov model of time series

We modelled the time series of swab data using a continuous-time HMM, as implemented in the R package msm (RRID:SCR_015500) (Jackson, 2011). Unobserved (true) states correspond to whether the child is carrying bacteria in their nasopharynx, and observed (emitted) states correspond to whether a positive swab was seen at each point. Transition probabilities between each state **Q** and the emission probabilities **E** are jointly estimated by maximum likelihood using the BOBYQA algorithm. We then constructed the most likely path through the unobserved states for each child using the Viterbi algorithm (Forney, 1973) with the observed data and estimated model parameters. Assuming that continuous occupation of the carried state corresponded to a single carriage episode, we calculated the duration for each such episode from the inferred true states.

### Processing genetic data

For each isolate with an inferred carriage duration (N = 2175) we extracted SNPs from the previously generated alignment against the ATCC 700669 genome (Chewapreecha et al., 2014b). Consequences of SNPs were annotated with VEP (RRID:SCR_007931), using a manually prepared reference (McLaren et al., 2010). A phylogenetic tree was generated from this alignment using FastTree (RRID:SCR_015501) under the GTR +gamma model (Price et al., 2009). The carriage duration was mapped on to this phylogeny using phytools (RRID:SCR_015502) (Revell, 2013). We then filtered the sites in the alignment to remove any where the major allele was an N, any sites with a minor allele frequency lower than 1%, and any sites where over 5% of calls were missing. This left 115210 sites for association testing and narrow-sense heritability estimation.

We counted 68M non-redundant k-mers with lengths 9–100 from the de novo assemblies of the genomes using a distributed string mining algorithm (Seth et al., 2014; Välimäki and Puglisi, 2012). We filtered out low frequency variants removing any k-mers with a minor allele frequency below 2%, leaving 17M for association testing.

We identified the presence of phage by performing a blastn of the de novo assemblies against a reference database of phage sequence (Croucher et al., 2016). If the length of the top hit was over 5000 we defined the isolate as having phage present (Figure 5).

### Transformation of carriage duration phenotype

As we aimed to fit a multiple linear regression model to the carriage duration y at each genetic locus k, we first ensured the data was appropriate for this model. The phenotype distribution was positively skewed, with an approximately exponential distribution (Figure 3). Residuals were therefore non-normally distributed, potentially reducing power (McCulloch, 2003). In the regression setting, a monotonic function can be applied to transform the response variable to avoid this problem. We took the natural logarithm of the carriage duration

$$\hat{𝐲}=\ln\,\left(𝐲\right)$$

which led to the residuals being much closer to being normally distributed (Figure 3). We applied the same transformation to child age, when it was used as a covariate in association.

### Estimation of heritability

We estimated broad sense heritability $H^{2}$ with the ANOVA-CPP method in the patherit R package (Mitov and Stadler, 2016), using a patristic distance cutoff of 0.04 (Figure 4). To test the effect of lineage genetics we used the patherit package to fit an Ornstein-Uhlenbeck model of the warped carriage duration along the phylogeny. We compared the likelihood of the full fit to that with no genetic effect on the trait ($\sigma_{G}^{2}=0$) using a likelihood ratio test (LRT) with one degree of freedom.

To estimate the SNP-based heritability $h_{\mathrm{SNP}}^{2}$ we applied a linear mixed model, which uses the genomic relatedness matrix (as calculated from SNPs passing filtering) as random effects. We used the implementation in warped-lmm (RRID:SCR_015503) (Fusi et al., 2014), which learns a monotonic transform as it fits the model to the data to ensure residuals are normally distributed (Figure 3). We therefore used the untransformed phenotype y as the input. Child age and whether previous carriage had occurred were included as covariates. We also estimated $h_{\mathrm{SNP}}^{2}$ using LDAK (RRID:SCR_015504) (Speed et al., 2012) with default settings, which gave an estimate of 0.437 (<1% difference from the warped-lmm estimate).

### Association of antimicrobial resistance and serotype with carriage duration

We encoded all 56 observed serotypes (including non-typables) and resistance to the six antibiotics as dummy variables. We used 6A/C as the reference level, as this had a mean carriage duration close to the grand mean in previous analysis. Orthogonal polynomial coding was used for the latter four antibiotics, where resistance could be intermediate or full. We then regressed this design matrix **X** was against the transformed carriage duration $\hat{𝐲}$. We removed three observations with low carriage lengths due to a delayed initial swab, and seven observations with leverages of one (Figure 3).

We performed variable selection using lasso regression (Efron et al., 2004), implemented in the R package glmnet (RRID:SCR_015505) (Friedman et al., 2010). We used leave-one-out cross-validation to choose a value for the $\ell_{1}$ penalty; the value one standard error above the minimum cross-validated error (Tibshirani et al., 2001) was selected (λ=0.033; Figure 4). The 20 predictors with non-zero coefficients in the model at this value of λ (Table 2) were used in a linear regression to calculate the multiple $R^{2}$, which corresponds to the proportion of variance explained by these predictors.

To estimate the variance components from serotype and resistance we used genomic partitioning (Yang et al., 2011), as implemented in LDAK. We used SNPs in the capsule locus to calculate a kinship matrix approximating the contribution from serotype variation. For antibiotic resistance we used SNPs in the *pbp* genes, *dyr* gene and ICE transposon to calculate a kinship matrix. Restricted maximum likelihood was used to estimate the variance explained by each of these components.

Capsule switch events had been previously identified by first reconstructing of the ancestral state of the serotype at each node through maximum parsimony (Chewapreecha et al., 2014a). For each node involving loss or gain of the capsule, those with at least one child being a tip were selected to find recent switches (all were capsule gain). The carriage duration of all unencapsulated children of the identified node were used as the null distribution to calculate an empirical p-value for the switched isolate. P-values were combined using Fisher's method (Rosenthal, 1978).

### Genome wide association of carriage duration

We used the linear mixed model implemented in fast-lmm (RRID:SCR_015506) (Lippert et al., 2011) to associate genetic elements with carriage duration, independent of overall lineage effects. We used the warped phenotype as the response, the kinship matrix (calculated from SNPs) as random effects, and variant presence, child age and previous carriage as fixed effects. For SNPs we used a Bonferroni correction with α<0.05 and an N of 92487 phylogenetically independent sites to derive a genome-wide significance cutoff of $p<5.4\times 10^{-7}$, and a suggestive significance cutoff (Lander and Kruglyak, 1995; Stranger et al., 2011) of $p=1.1\times 10^{-4}$. We tested pairwise LD between the significant SNPs by calculating the $R^{2}$ between them. We removed those with $R^{2}\;>\;0.2$, assuming these represented the same underlying signal, to define the significant loci. To perform conditional analysis we used the pattern of the most significant SNP as a fixed-effect, removed it from the test and kinship estimation, and re-ran the mixed model on all other sites.

For k-mers we counted 5254876 phylogenetically independent sites, giving a genome wide significance cutoff of $9.5\times 10^{-9}$. We used blastn (RRID:SCR_001598) with default settings to map the significant k-mers to seven reference genomes (ATCC 700669, INV104B, OXC141, SPNA45, Taiwan19F, TIGR4 and NT_110_58), and the possible Tn*916* sequences (Croucher et al., 2011).

To search for variants with some level of lineage independence we used SEER (RRID:SCR_015499) (Lees et al., 2016). To correct for population structure we used the patristic distances from the phylogenetic tree as the kinship matrix, which we then projected into 30 dimensions using metric multidimensional scaling. The coordinates of the samples in this space were used as covariates in SEER’s linear regression. We performed association tests on SNPs and k-mers with MAF > 1% using multiple linear regression, and report the top hits with $p<10^{-14}$. Significant k-mers were mapped as above.

### Code and data availability

All code used for analysis is available on github, along with inferred carriage duration for each sample (Lees, 2017). A copy is archived at https://github.com/elifesciences-publications/carriage-duration.

## Additional files

### Major datasets

The following previously published datasets were used:

Chewapreecha CHarris SRCroucher NJTurner CMarttinen PCheng LPessia AAanensen DMMather AEPage AJSalter SJHarris DNosten FGoldblatt DCorander JParkhill JTurner PBentley SD2014Dense genomic sampling identifies highways of pneumococcal recombinationhttps://www.ncbi.nlm.nih.gov/sra/?term=ERP000435Publicly available in the NCBI Sequencing Read Archive (SRA) (accession no. ERP000435)

Chewapreecha CHarris SRCroucher NJTurner CMarttinen PCheng LPessia AAanensen DMMather AEPage AJSalter SJHarris DNosten FGoldblatt DCorander JParkhill JTurner PBentley SD2014Dense genomic sampling identifies highways of pneumococcal recombinationhttps://www.ncbi.nlm.nih.gov/sra/?term=ERP000483Publicly available in the NCBI Sequencing Read Archive (SRA) (accession no. ERP000483)

Chewapreecha CHarris SRCroucher NJTurner CMarttinen PCheng LPessia AAanensen DMMather AEPage AJSalter SJHarris DNosten FGoldblatt DCorander JParkhill JTurner PBentley SD2014Dense genomic sampling identifies highways of pneumococcal recombinationhttps://www.ncbi.nlm.nih.gov/sra/?term=ERP000485Publicly available in the NCBI Sequencing Read Archive (SRA) (accession no. ERP000485)

Chewapreecha CHarris SRCroucher NJTurner CMarttinen PCheng LPessia AAanensen DMMather AEPage AJSalter SJHarris DNosten FGoldblatt DCorander JParkhill JTurner PBentley SD2014Dense genomic sampling identifies highways of pneumococcal recombinationhttps://www.ncbi.nlm.nih.gov/sra/?term=ERP000487Publicly available in the NCBI Sequencing Read Archive (SRA) (accession no. ERP000487)

Chewapreecha CHarris SRCroucher NJTurner CMarttinen PCheng LPessia AAanensen DMMather AEPage AJSalter SJHarris DNosten FGoldblatt DCorander JParkhill JTurner PBentley SD2014Dense genomic sampling identifies highways of pneumococcal recombinationhttps://www.ncbi.nlm.nih.gov/sra/?term=ERP000598Publicly available in the NCBI Sequencing Read Archive (SRA) (accession no. ERP000598)

Chewapreecha CHarris SRCroucher NJTurner CMarttinen PCheng LPessia AAanensen DMMather AEPage AJSalter SJHarris DNosten FGoldblatt DCorander JParkhill JTurner PBentley SD2014Dense genomic sampling identifies highways of pneumococcal recombinationhttps://www.ncbi.nlm.nih.gov/sra/?term=ERP000599Publicly available in the NCBI Sequencing Read Archive (SRA) (accession no. ERP000599)

## References

[bib1] Abdullahi O, Karani A, Tigoi CC, Mugo D, Kungu S, Wanjiru E, Jomo J, Musyimi R, Lipsitch M, Scott JA (2012a). Rates of acquisition and clearance of pneumococcal serotypes in the nasopharynges of children in Kilifi District, Kenya. The Journal of Infectious Diseases.

[bib2] Abdullahi O, Karani A, Tigoi CC, Mugo D, Kungu S, Wanjiru E, Jomo J, Musyimi R, Lipsitch M, Scott JA (2012b). The prevalence and risk factors for pneumococcal colonization of the nasopharynx among children in Kilifi District, Kenya. PLoS One.

[bib3] Akaike H (1974). A new look at the statistical model identification. IEEE Transactions on Automatic Control.

[bib4] Anderson TJC, Williams JT, Nair S, Sudimack D, Barends M, Jaidee A, Price RN, Nosten F (2010). Inferred relatedness and heritability in malaria parasites. Proceedings of the Royal Society B: Biological Sciences.

[bib5] Auranen K, Mehtälä J, Tanskanen A, S Kaltoft M (2010). Between-strain competition in acquisition and clearance of pneumococcal carriage--epidemiologic evidence from a longitudinal study of day-care children. American Journal of Epidemiology.

[bib6] Bille E, Zahar JR, Perrin A, Morelle S, Kriz P, Jolley KA, Maiden MC, Dervin C, Nassif X, Tinsley CR (2005). A chromosomally integrated bacteriophage in invasive meningococci. The Journal of Experimental Medicine.

[bib7] Bille E, Ure R, Gray SJ, Kaczmarski EB, McCarthy ND, Nassif X, Maiden MC, Tinsley CR (2008). Association of a bacteriophage with meningococcal disease in young adults. PLoS ONE.

[bib8] Bogaardt C, van Tonder AJ, Brueggemann AB (2015). Genomic analyses of pneumococci reveal a wide diversity of bacteriocins - including pneumocyclicin, a novel circular bacteriocin. BMC Genomics.

[bib9] Brueggemann AB, Griffiths DT, Meats E, Peto T, Crook DW, Spratt BG (2003). Clonal relationships between invasive and carriage *Streptococcus pneumoniae* and serotype- and clone-specific differences in invasive disease potential. The Journal of Infectious Diseases.

[bib10] Chaguza C, Andam CP, Harris SR, Cornick JE, Yang M, Bricio-Moreno L, Kamng'ona AW, Parkhill J, French N, Heyderman RS, Kadioglu A, Everett DB, Bentley SD, Hanage WP (2016). Recombination in *Streptococcus pneumoniae* Lineages Increase with Carriage Duration and Size of the Polysaccharide Capsule. mBio.

[bib11] Chen PE, Shapiro BJ (2015). The advent of genome-wide association studies for bacteria. Current Opinion in Microbiology.

[bib12] Chewapreecha C, Harris SR, Croucher NJ, Turner C, Marttinen P, Cheng L, Pessia A, Aanensen DM, Mather AE, Page AJ, Salter SJ, Harris D, Nosten F, Goldblatt D, Corander J, Parkhill J, Turner P, Bentley SD (2014a). Dense genomic sampling identifies highways of pneumococcal recombination. Nature Genetics.

[bib13] Chewapreecha C, Marttinen P, Croucher NJ, Salter SJ, Harris SR, Mather AE, Hanage WP, Goldblatt D, Nosten FH, Turner C, Turner P, Bentley SD, Parkhill J (2014b). Comprehensive identification of single nucleotide polymorphisms associated with beta-lactam resistance within pneumococcal mosaic genes. PLoS Genetics.

[bib14] Cobey S, Lipsitch M (2012). Niche and neutral effects of acquired immunity permit coexistence of pneumococcal serotypes. Science.

[bib15] Croucher NJ, Walker D, Romero P, Lennard N, Paterson GK, Bason NC, Mitchell AM, Quail MA, Andrew PW, Parkhill J, Bentley SD, Mitchell TJ (2009). Role of conjugative elements in the evolution of the multidrug-resistant pandemic clone Streptococcus pneumoniaeSpain23F ST81. Journal of Bacteriology.

[bib16] Croucher NJ, Harris SR, Fraser C, Quail MA, Burton J, van der Linden M, McGee L, von Gottberg A, Song JH, Ko KS, Pichon B, Baker S, Parry CM, Lambertsen LM, Shahinas D, Pillai DR, Mitchell TJ, Dougan G, Tomasz A, Klugman KP, Parkhill J, Hanage WP, Bentley SD (2011). Rapid pneumococcal evolution in response to clinical interventions. Science.

[bib17] Croucher NJ, Coupland PG, Stevenson AE, Callendrello A, Bentley SD, Hanage WP (2014a). Diversification of bacterial genome content through distinct mechanisms over different timescales. Nature Communications.

[bib18] Croucher NJ, Hanage WP, Harris SR, McGee L, van der Linden M, de Lencastre H, Sá-Leão R, Song JH, Ko KS, Beall B, Klugman KP, Parkhill J, Tomasz A, Kristinsson KG, Bentley SD (2014b). Variable recombination dynamics during the emergence, transmission and 'disarming' of a multidrug-resistant pneumococcal clone. BMC Biology.

[bib19] Croucher NJ, Mostowy R, Wymant C, Turner P, Bentley SD, Fraser C (2016). Horizontal DNA Transfer Mechanisms of Bacteria as Weapons of Intragenomic Conflict. PLOS Biology.

[bib20] Dawid S, Roche AM, Weiser JN (2007). The blp bacteriocins of Streptococcus pneumoniae mediate intraspecies competition both in vitro and in vivo. Infection and Immunity.

[bib21] DeBardeleben HK, Lysenko ES, Dalia AB, Weiser JN (2014). Tolerance of a phage element by Streptococcus pneumoniae leads to a fitness defect during colonization. Journal of Bacteriology.

[bib22] Duplessis M, Moineau S (2001). Identification of a genetic determinant responsible for host specificity in Streptococcus thermophilus bacteriophages. Molecular Microbiology.

[bib23] Earle SG, Wu CH, Charlesworth J, Stoesser N, Gordon NC, Walker TM, Spencer CCA, Iqbal Z, Clifton DA, Hopkins KL, Woodford N, Smith EG, Ismail N, Llewelyn MJ, Peto TE, Crook DW, McVean G, Walker AS, Wilson DJ (2016). Identifying lineage effects when controlling for population structure improves power in bacterial association studies. Nature Microbiology.

[bib24] Efron B, Hastie T, Johnstone I, Tibshirani R (2004). Least angle regression. Annals of Statistics.

[bib25] Enright MC, Spratt BG (1998). A multilocus sequence typing scheme for Streptococcus pneumoniae: identification of clones associated with serious invasive disease. Microbiology.

[bib26] Forney GD (1973). The viterbi algorithm. Proceeding of the IEEE.

[bib27] Fraser C, Lythgoe K, Leventhal GE, Shirreff G, Hollingsworth TD, Alizon S, Bonhoeffer S (2014). Virulence and pathogenesis of HIV-1 infection: an evolutionary perspective. Science.

[bib28] Friedman J, Hastie T, Tibshirani R (2010). Regularization Paths for Generalized Linear Models via Coordinate Descent. Journal of Statistical Software.

[bib29] Fusi N, Lippert C, Lawrence ND, Stegle O (2014). Warped linear mixed models for the genetic analysis of transformed phenotypes. Nature Communications.

[bib30] Gritzfeld JF, Cremers AJ, Ferwerda G, Ferreira DM, Kadioglu A, Hermans PW, Gordon SB (2014). Density and duration of experimental human pneumococcal carriage. Clinical Microbiology and Infection.

[bib31] Hanage WP, Fraser C, Tang J, Connor TR, Corander J (2009). Hyper-recombination, diversity, and antibiotic resistance in pneumococcus. Science.

[bib32] Hardin G (1960). The competitive exclusion principle. Science.

[bib33] Hathaway LJ, Brugger SD, Morand B, Bangert M, Rotzetter JU, Hauser C, Graber WA, Gore S, Kadioglu A, Mühlemann K (2012). Capsule type of Streptococcus pneumoniae determines growth phenotype. PLoS Pathogens.

[bib34] Hebiri M, Lederer JC (2012). How correlations influence lasso prediction. arXiv.

[bib35] Hill PC, Townend J, Antonio M, Akisanya B, Ebruke C, Lahai G, Greenwood BM, Adegbola RA (2010). Transmission of *Streptococcus pneumoniae* in rural Gambian villages: a longitudinal study. Clinical Infectious Diseases.

[bib36] Högberg L, Geli P, Ringberg H, Melander E, Lipsitch M, Ekdahl K (2007). Age- and serogroup-related differences in observed durations of nasopharyngeal carriage of penicillin-resistant pneumococci. Journal of Clinical Microbiology.

[bib37] Iannelli F, Oggioni MR, Pozzi G (2002). Allelic variation in the highly polymorphic locus pspC of Streptococcus pneumoniae. Gene.

[bib38] Jackson CH (2011). Multi-state models for panel data: The **msm** package for *R*. Journal of Statistical Software.

[bib39] Jedrzejas MJ, Lamani E, Becker RS (2001). Characterization of selected strains of pneumococcal surface protein A. Journal of Biological Chemistry.

[bib40] Kadioglu A, Weiser JN, Paton JC, Andrew PW (2008). The role of Streptococcus pneumoniae virulence factors in host respiratory colonization and disease. Nature Reviews Microbiology.

[bib41] Khan MN, Coleman JR, Vernatter J, Varshney AK, Dufaud C, Pirofski LA (2014). An ahemolytic pneumolysin of Streptococcus pneumoniae manipulates human innate and CD4⁺ T-cell responses and reduces resistance to colonization in mice in a serotype-independent manner. Journal of Infectious Diseases.

[bib42] Kono M, Zafar MA, Zuniga M, Roche AM, Hamaguchi S, Weiser JN (2016). Single Cell Bottlenecks in the Pathogenesis of Streptococcus pneumoniae. PLOS Pathogens.

[bib43] Lander E, Kruglyak L (1995). Genetic dissection of complex traits: guidelines for interpreting and reporting linkage results. Nature Genetics.

[bib44] Lee SH, Wray NR, Goddard ME, Visscher PM (2011). Estimating missing heritability for disease from genome-wide association studies. The American Journal of Human Genetics.

[bib45] Lees JA, Vehkala M, Välimäki N, Harris SR, Chewapreecha C, Croucher NJ, Marttinen P, Davies MR, Steer AC, Tong SY, Honkela A, Parkhill J, Bentley SD, Corander J (2016). Sequence element enrichment analysis to determine the genetic basis of bacterial phenotypes. Nature Communications.

[bib46] Lees JA (2017). GitHub.

[bib47] Lehtinen S, Blanquart F, Croucher NJ, Turner P, Lipsitch M, Fraser C (2017). Evolution of antibiotic resistance is linked to any genetic mechanism affecting bacterial duration of carriage. PNAS.

[bib48] Lippert C, Listgarten J, Liu Y, Kadie CM, Davidson RI, Heckerman D (2011). FaST linear mixed models for genome-wide association studies. Nature Methods.

[bib49] Lipsitch M, O'Hagan JJ (2007). Patterns of antigenic diversity and the mechanisms that maintain them. Journal of the Royal Society Interface.

[bib50] Lockhart R, Taylor J, Tibshirani RJ, Tibshirani R (2014). A SIGNIFICANCE TEST FOR THE LASSO. The Annals of Statistics.

[bib51] Loeffler JM, Fischetti VA (2006). Lysogeny of Streptococcus pneumoniae with MM1 phage: improved adherence and other phenotypic changes. Infection and Immunity.

[bib52] Manolio TA, Collins FS, Cox NJ, Goldstein DB, Hindorff LA, Hunter DJ, McCarthy MI, Ramos EM, Cardon LR, Chakravarti A, Cho JH, Guttmacher AE, Kong A, Kruglyak L, Mardis E, Rotimi CN, Slatkin M, Valle D, Whittemore AS, Boehnke M, Clark AG, Eichler EE, Gibson G, Haines JL, Mackay TF, McCarroll SA, Visscher PM (2009). Finding the missing heritability of complex diseases. Nature.

[bib53] Maródi L (2006). Neonatal innate immunity to infectious agents. Infection and Immunity.

[bib54] McCool TL, Cate TR, Moy G, Weiser JN (2002). The immune response to pneumococcal proteins during experimental human carriage. The Journal of Experimental Medicine.

[bib55] McCulloch CE (2003). Chapter 4: generalized linear mixed models (GLMMs). Generalized Linear Mixed Models.

[bib56] McLaren W, Pritchard B, Rios D, Chen Y, Flicek P, Cunningham F (2010). Deriving the consequences of genomic variants with the Ensembl API and SNP Effect Predictor. Bioinformatics.

[bib57] Melegaro A, Gay NJ, Medley GF (2004). Estimating the transmission parameters of pneumococcal carriage in households. Epidemiology and Infection.

[bib58] Melegaro A, Choi Y, Pebody R, Gay N (2007). Pneumococcal carriage in United Kingdom families: estimating serotype-specific transmission parameters from longitudinal data. American Journal of Epidemiology.

[bib59] Melin M, Trzciński K, Meri S, Käyhty H, Väkeväinen M (2010). The capsular serotype of Streptococcus pneumoniae is more important than the genetic background for resistance to complement. Infection and Immunity.

[bib60] Mitov V, Stadler T (2016). The heritability of pathogen traits - definitions and estimators. biorxiv.

[bib61] O'Brien KL, Wolfson LJ, Watt JP, Henkle E, Deloria-Knoll M, McCall N, Lee E, Mulholland K, Levine OS, Cherian T (2009). Burden of disease caused by Streptococcus pneumoniae in children younger than 5 years: global estimates. The Lancet.

[bib62] Pericone CD, Overweg K, Hermans PW, Weiser JN (2000). Inhibitory and bactericidal effects of hydrogen peroxide production by Streptococcus pneumoniae on other inhabitants of the upper respiratory tract. Infection and Immunity.

[bib63] Power RA, Parkhill J, de Oliveira T (2017). Microbial genome-wide association studies: lessons from human GWAS. Nature Reviews Genetics.

[bib64] Price MN, Dehal PS, Arkin AP (2009). FastTree: computing large minimum evolution trees with profiles instead of a distance matrix. Molecular Biology and Evolution.

[bib65] Read TD, Massey RC (2014). Characterizing the genetic basis of bacterial phenotypes using genome-wide association studies: a new direction for bacteriology. Genome Medicine.

[bib66] Revell LJ (2013). Two new graphical methods for mapping trait evolution on phylogenies. Methods in Ecology and Evolution.

[bib67] Romero P, Croucher NJ, Hiller NL, Hu FZ, Ehrlich GD, Bentley SD, García E, Mitchell TJ (2009). Comparative genomic analysis of ten Streptococcus pneumoniae temperate bacteriophages. Journal of Bacteriology.

[bib68] Rosenthal R (1978). Combining results of independent studies. Psychological Bulletin.

[bib69] Seth S, Välimäki N, Kaski S, Honkela A (2014). Exploration and retrieval of whole-metagenome sequencing samples. Bioinformatics.

[bib70] Shapiro ED, Austrian R (1994). Serotypes responsible for invasive Streptococcus pneumoniae infections among children in Connecticut. Journal of Infectious Diseases.

[bib71] Snelson E, Ghahramani Z, Rasmussen CE, Thrun S, Saul L. K, Schölkopf P. B (2004). Warped gaussian processes. Advances in Neural Information Processing Systems.

[bib72] Speed D, Hemani G, Johnson MR, Balding DJ (2012). Improved heritability estimation from genome-wide SNPs. The American Journal of Human Genetics.

[bib73] Stranger BE, Stahl EA, Raj T (2011). Progress and promise of genome-wide association studies for human complex trait genetics. Genetics.

[bib74] Tibshirani R, Walther G, Hastie T (2001). Estimating the number of clusters in a data set via the gap statistic. Journal of the Royal Statistical Society: Series B.

[bib75] Trzciński K, Li Y, Weinberger DM, Thompson CM, Cordy D, Bessolo A, Malley R, Lipsitch M (2015). Effect of serotype on pneumococcal competition in a mouse colonization model. mBio.

[bib76] Turner P, Turner C, Jankhot A, Helen N, Lee SJ, Day NP, White NJ, Nosten F, Goldblatt D (2012). A longitudinal study of Streptococcus pneumoniae carriage in a cohort of infants and their mothers on the Thailand-Myanmar border. PLoS ONE.

[bib77] Turner C, Turner P, Carrara V, Burgoine K, Tha Ler Htoo S, Watthanaworawit W, Day NP, White NJ, Goldblatt D, Nosten F (2013a). High rates of pneumonia in children under two years of age in a South East Asian refugee population. PLoS One.

[bib78] Turner P, Turner C, Jankhot A, Phakaudom K, Nosten F, Goldblatt D (2013b). Field evaluation of culture plus latex sweep serotyping for detection of multiple pneumococcal serotype colonisation in infants and young children. PLoS One.

[bib79] Visscher PM, Hill WG, Wray NR (2008). Heritability in the genomics era — concepts and misconceptions. Nature Reviews Genetics.

[bib80] Välimäki N, Puglisi S, Raphael B, Tang J (2012). Distributed string mining for High-Throughput sequencing data. Algorithms in Bioinformatics SE - 35, Lecture Notes in Computer Science.

[bib81] Weinberger DM, Dagan R, Givon-Lavi N, Regev-Yochay G, Malley R, Lipsitch M (2008). Epidemiologic evidence for serotype-specific acquired immunity to pneumococcal carriage. The Journal of Infectious Diseases.

[bib82] Weinberger DM, Trzciński K, Lu YJ, Bogaert D, Brandes A, Galagan J, Anderson PW, Malley R, Lipsitch M (2009). Pneumococcal capsular polysaccharide structure predicts serotype prevalence. PLoS Pathogens.

[bib83] Weinberger DM, Harboe ZB, Flasche S, Scott JA, Lipsitch M (2011). Prediction of serotypes causing invasive pneumococcal disease in unvaccinated and vaccinated populations. Epidemiology.

[bib84] Yang J, Manolio TA, Pasquale LR, Boerwinkle E, Caporaso N, Cunningham JM, de Andrade M, Feenstra B, Feingold E, Hayes MG, Hill WG, Landi MT, Alonso A, Lettre G, Lin P, Ling H, Lowe W, Mathias RA, Melbye M, Pugh E, Cornelis MC, Weir BS, Goddard ME, Visscher PM (2011). Genome partitioning of genetic variation for complex traits using common SNPs. Nature Genetics.

